# Prevalence of ADHD in Adults: An Umbrella Review of International Studies

**DOI:** 10.1192/j.eurpsy.2024.708

**Published:** 2024-08-27

**Authors:** G. Ayano, L. Tsegay, Y. Gizachew, M. Necho, K. Yohannes, S. Demelash, T. Anbesaw, R. Alati

**Affiliations:** ^1^Population Health, Curtin University, Perth, Australia; ^2^Aksum University, Aksum; ^3^Bethel Medical College, Addis Ababa; ^4^Wollo University, Amhara, Ethiopia; ^5^SWEDESD, Department of Women’s and Children’s Health, Uppsala University, Upsala, Sweden; ^6^Ethiopian Public Health Insitute, Addis Ababa, Ethiopia

## Abstract

**Introduction:**

Attention deficit hyperactivity disorder (ADHD) is a neurodevelopmental disorder commonly diagnosed in school-age children. However, it can affect individuals of all age groups. This study aimed to provide a comprehensive analysis of the prevalence of ADHD in adults by conducting an umbrella review of systematic reviews and meta-analyses.

**Objectives:**

To provide a comprehensive synthesis of published evidence on the prevalence of Attention Deficit Hyperactivity Disorder (ADHD) in adults through an umbrella review of systematic reviews and meta-analyses, with the aim of highlighting the significance of addressing and managing ADHD in the adult population.

**Methods:**

To conduct this study, we adhered to the guidelines outlined in the Preferred Reporting Items for Systematic Review and Meta-Analysis (PRISMA). We systematically searched databases such as PsychINFO, Web of Science, PubMed, and Scopus to identify relevant studies. Our review protocol was registered with PROSPERO (registration number: CRD42023389704). The quality of the studies included in our analysis was assessed using the A Measurement Tool to Assess Systematic Reviews (AMSTAR). For the purpose of conducting a meta-analysis, we employed a random-effects model.

**Results:**

Our umbrella review examined findings from five systematic reviews that encompassed data from 57 unique international primary studies undertaken between 2009 and 2021. These studies involved a total of 21,142,129 adult participants. The meta-analysis, employing an inverse variance-weighted random effect model, yielded a pooled prevalence estimate for ADHD in adults of 3.10% (95% confidence interval: 2.60%–3.60%). Regarding ADHD subtypes, our analysis revealed that ADHD-I (inattentive type) remained the most prevalent among adults, followed by ADHD-HI (hyperactive type) and ADHD-C (combined type).

**Conclusions:**

Our results underscore the relatively high prevalence of ADHD among adults, with ADHD-I emerging as the most common subtype. These findings emphasize the need for proactive measures to prevent, mitigate, identify, and effectively manage ADHD in the adult population.

**Disclosure of Interest:**

None Declared

